# Lysine‐specific demethylase 1 aggravated oxidative stress and ferroptosis induced by renal ischemia and reperfusion injury through activation of TLR4/NOX4 pathway in mice

**DOI:** 10.1111/jcmm.17444

**Published:** 2022-06-30

**Authors:** Ruikang Feng, Yufeng Xiong, Yourong Lei, Qin Huang, Hao Liu, Xiaojie Zhao, Zhiyuan Chen, Hui Chen, Xiuheng Liu, Lei Wang, Xiaodong Weng

**Affiliations:** ^1^ Department of Urology Renmin Hospital of Wuhan University Wuhan China; ^2^ Department of infection prevention and control Renmin Hospital of Wuhan University Wuhan China; ^3^ Department of Obstetrics and Gynecology Renmin Hospital of Wuhan University Wuhan China

**Keywords:** ferroptosis, ischaemia and reperfusion injury, LSD1, oxidative stress

## Abstract

Acute kidney injury (AKI) is mainly caused by renal ischaemia reperfusion injury (IRI). Lots of evidence suggests that ferroptosis and oxidative stress play the vital role in renal IRI. However, the specific mechanism of renal IRI has not been fully elucidated. lysine‐specific demethylase 1 (LSD1) has been shown to regulate the pathogenesis of kidney disease. In this study, we firstly found that LSD1 was positively related to renal IRI. TCP, a classical LSD1 inhibitor, could alleviate tissue damage induced by renal IRI. Inhibition of LSD1 with either TCP or LSD1 knockdown could alleviate ferroptosis and oxidative stress caused by IRI both in vivo and in vitro. Furthermore, the results showed that suppression of LSD1 decreased the expression of TLR4/NOX4 pathway in HK‐2 cells subjected to H/R. With the si‐RNA against TLR4 or NOX4, it showed that the silence of TLR4/NOX4 reduced oxidative stress and ferroptosis in vitro. Moreover, to demonstrate the crucial role of TLR4/NOX4, TLR4 reduction, mediated by inhibition of LSD1, was compensated through delivering the adenovirus carrying TLR4 in vitro. The results showed that the compensation of TLR4 blunted the alleviation of oxidative stress and ferroptosis, induced by LSD1 inhibition. Further study showed that LSD1 activates TLR4/NOX4 pathway by reducing the enrichment of H3K9me2 in the TLR4 promoter region. In conclusion, our results demonstrated that LSD1 inhibition blocked ferroptosis and oxidative stress caused by renal IRI through the TLR4/NOX4 pathway, indicating that LSD1 could be a potential therapeutic target for renal IRI.

## 1 INTRODUCTION

Acute kidney injury (AKI) is a clinical syndrome, defined by a sudden decrease in glomerular filtration.^1^ In Clinics, AKI is mainly resulted from renal ischaemia reperfusion injury (IRI),^2^ which is usually caused by hypovolemic shock, surgery and transplantation.^3^ Renal IRI can induce inflammatory cells to release proteases and pro‐inflammatory cytokines, block capillaries around renal tubules, and produce reactive oxygen species (ROS), which can induce the death of renal tubular cells and lead to structural and functional damage of renal tubules.^4^ However, the mechanism of renal IRI has not been totally elucidated.^5^ Therefore, it is urgent to explore the potential mechanism and effective treatment of renal IRI.

The epigenetic state determined by genotype may be the direct cause of the changes in gene activity.^6^ Numerous reports have indicated that epigenetic process is involved in the pathogenesis of AKI.^7^ Lysine specific demethylase 1 (LSD1), which was able to remove the mono‐ or dimethyl‐lysine 9 of histone H3 (H3K9me1/2), could regulate downstream gene expression and thus affect cellular process.^8^, ^9^ Previous studies found that LSD1 played a vital role in myocardial and cerebral IRI.^10^, ^11^ However, it is still unknown that whether LSD1 plays a role in renal IRI.

Recently, several novel cell death processes with unique regulatory pathways have been discovered.^12^ Ferroptosis, characterized by the iron‐dependent accumulation of lipid hydroperoxides to lethal levels, is a form of regulated cell death.^13^ The biological and pathological function of ferroptosis has been elucidated, including its effect on tumour suppression and organ damage.^14^ It has been reported that the role of ferroptosis on AKI. It was indicated that dysfunction of the key ferroptosis‐surveilling systems hypersensitized mice to tubular necrosis during AKI.^15^ Also, others found that ferroptosis was the vital mechanism of cell death in folic acid induced AKI.^16^ Nevertheless, the mechanism of ferroptosis during renal IRI has not been fully elucidated.

Oxidative stress plays a key role in the apoptosis of renal tubular cells, especially in the process of renal IRI.^17^, ^18^ Reactive oxygen species (ROS) can induce cell apoptosis in various condition through directly causing cell damage or acting as signal transduction molecules. It has been demonstrated that LSD1 mediated oxidative stress to active AKT pathway.^19^ However, how LSD1 regulating oxidative stress during renal IRI is still unknown. In our study, we focused on investigating whether LSD1 inhibition could regulate IRI‐induced AKI. Furthermore, the present study investigated the relationship between LSD1 and oxidative stress, and its potential mechanism.

## 2 METHODS

### 2.1 Animals groups and treatments

Adult male C57BL6 (C57) mice (8–12 weeks, 20–25 g) were purchased from the Animal Experiment Center of Wuhan University. This experiment was approved by the Ethics Committee of Renmin Hospital of Wuhan University, and the procedures were carried out adhere to the principles of Animal Care of Wuhan University (Wuhan, China). The renal IRI model was conducted in the Animal Experiment Center of Wuhan University as follows. The mice were anaesthetised with 50 mg/kg pentobarbital (i.p.) and put on thermostatic operating table to maintain the temperature around 37°C. The right kidney was excised, and then, a microvascular clip was employed to clamp the pedicle of the left kidney for 30 min. All mice were euthanized and sacrificed at reperfusion 0, 12, or 24 h.

All 64 mice were randomly divided into various groups by different treatments (*n* = 8). In sham group, after the right kidney excised, the left renal pedicles were without any treatment. In IRI group, the pedicle of the left kidney was clamped for 30 min followed by various reperfusion periods (6, 12, 24 h). To study the effects of LSD1, TCP (MedChemExpress) was injected intraperitoneally at different doses (2.5, 5, 10 mg/kg) before IRI model establishment, once a day for 1 week. TCP powder was dissolved in dimethyl sulfoxide (DMSO). In the vehicle control group, equal amount of DMSO was injected intraperitoneally.

### 2.2 Cell culture and H/R model

The human renal tubular epithelial cells (HK‐2) were obtained by the American Type Culture Collection. The cells were cultured in DMEM (Invitrogen) containing 10% foetal bovine serum (Gibco) and 1% penicillin and streptomycin under constant‐temperature incubator (37°C, 5% CO_2_, 21% O_2_). To establish H/R model, HK‐2 cells were firstly placed in medium without nutrients (glucose and serum) under hypoxic incubator (94% N_2_, 5% CO_2_, and 1% O_2_) for 12 h, and then, it was replaced in constant‐temperature incubator (37°C, 5% CO_2_, 21% O_2_) for 2, 4 and 6 h. The cells in control group were cultured in constant‐temperature incubator (37°C, 5% CO_2_, 21% O_2_).

### 2.3 Real‐time quantitative reverse transcription‐polymerase chain reaction

Total RNA was extracted from the cells or frozen renal tissue using RNAiso plus (Takara Biotechnology Company) according to the instructions of the reagent manufacturer, and then, primescript™ RT Kit (Takara Biotechnology Company) was used to reverse transcribe it into cDNA. The real‐time quantitative PCR procedure was carried out using ABIViiA7DX system. GAPDH was used for experimental reference. RT‐PCR primers designed for specific target genes are synthesized by Takara biotech (listed below).

H‐LSD1: 5′‐ GTGGTAACAGGTCTTGGAGGGA‐3′ (F)
5′‐ CAGCTTGTCCGTTGGCTTCAT‐3′ (R)
H‐GAPDH: 5′‐ GTCAAGGCTGAGAACGGGAA‐3′ (F)
5′‐ AAATGAGCCCCAGCCTTCTC‐3′ (R)
M‐LSD1: 5′‐ CCACCGAGTTCACAGTTACTTAGAG‐3′ (F)
5′‐ TAGCAACTCGTCCACCTACTCG‐3′ (R)
M‐GAPDH: 5′‐ TGATGGGTGTGAACCACGAG‐3′ (F)
5′‐ AGTGATGGCATGGACTGTGG ‐3′ (R)

### 2.4 Western blot

Samples were collected and snap‐frozen in liquid nitrogen. Then they were used to extract total proteins. The Bicinchoninic Acid (BCA) method was to quantify protein levels. Then, protein samples were separated on sodium dodecyl sulphate‐polyacrylamide gel electrophoresis (SDS‐PAGE) gels and then transferred to a polyvinylidene difluoride (PVDF) membrane. Subsequently, PVDF membranes were blocked with 5% non‐fat milk for 2 hours and then incubated at 4°C overnight with specific antibodies against LSD1 (#129195, Abcam), 4‐Hydroxynonenal (4‐HNE) (#46545, Abcam), TLR4 (#13556, Abcam), NOX4(#293072, Santa Cruz), ASCL4 (A14439, ABclonal), GPX4(#125066, Abcam), FSP1(#197896, Abcam), H3K9me2(#176882, Abcam) and β‐actin (#BA2305, Boster Biological Technology). After these, they were washed with PBST and incubated with secondary antibodies at room temperature. Specific bands were detected by ECL™ (Beijing Pierce Biotechnology), and the densities were quantified using Image J software.

### 2.5 Renal function

1 ml blood samples were collected and renal function was measured by creatinine and blood urea nitrogen commercial kits (Nanjing Jiancheng Bioengineering Institute, C011‐2‐1 and C013‐2‐1) according to the manufacturer's instructions.

### 2.6 Histological staining and immunohistochemistry

The kidney tissues of mice were fixed by formalin and embedded in paraffin; then, the tissues were cut into 4 μm‐thick section. Then, the slides were dewaxed, hydrated and then stained with haematoxylin and eosin (H&E). By analysing the proportion of tubular necrosis, dilatation, tubular formation and nuclear consolidation, tubular injury was scored as 0–4: 0, no injury; 1, 1%–25%; 2, 26%–50%; 3, 51%–75%; and 4, 76%–100%. Histological injury scores were graded by two different and experienced pathologists to assess morphological changes who were unaware of the groups.

Immunohistochemical staining was used to detect LSD1 localization. The sections were firstly incubated with primary antibody against LSD1 (1:100) at 4°C overnight, followed by secondary antibody at room temperature for 30 min. To evaluate the staining intensity, all fields (×400) were photographed randomly. For quantification, the relative mean integrated optical density (IOD) of each group was analysed using Image‐Pro Plus 7.0 (Media Cybernetics).

### 2.7 Superoxide dismutase and malondialdehyde

According to the manufacturer's instructions, the activity levels of Superoxide dismutase (SOD) (WST‐1 method) and malondialdehyde (MDA) (TBA method) in renal tissue lysates or HK‐2 cells were measured using the commercial kit (Nanjing Jiancheng Bioengineering Institute, A001‐3‐2 and A003‐1‐2).

### 2.8 The measurement of glutathione and iron level

The cells or kidney tissues were collected and homogenized with phosphate buffered saline (PBS). The level of normalized iron concentration (Fe^2+^ level) was detection according to the Iron Assay Kit (Abcam) instructions. The level of glutathione (GSH) in cell or kidney tissues was according to the commercial Kit (Nanjing Jiancheng Bioengineering Institute).

### 2.9 Cell viability

Cell viability was measured using CCK‐8 assay kit according to the instructions (Beyotime) at 450 nm using a microplate reader.

### 2.10 Small interfering RNA

LSD1, TLR4 and NOX4 specific Small interfering RNA (siRNA) and non‐targeted siRNA were designed and synthesized by GenePharma. HK‐2 cells were transfected with 100 nM of siRNA and Lipofectamine 3000 (Invitrogen) in antibiotic‐free medium. Western blot was used to confirm the effect of siRNA transfection.

### 2.11 Adenoviral infection

Overexpression of TLR4 was established via infection of cells with adenovirus in DMEM/F12 for 6 h, without any serum, penicillin and streptomycin. Then, they were replaced with the medium containing 10% foetal bovine serum and cultured for 72 h.

### 2.12 Determination of intracellular reactive oxygen species

We used commercial 2=,7=‐dichlorodihydrofluorescein (DCFH) diacetate molecular probes (Sigma) to measure intracellular ROS levels. In brief, HK‐2 cells in six‐well plates were incubate with 2 ml of 10 μM DCFH diacetate probes for 30 min at 37°C in the dark. Cells were washed three times with serum‐free cell medium. Intracellular ROS can oxidize nonfluorescent DCFH to dichlorofluorescein (DCF). The level of intracellular ROS can be determined by detecting the fluorescence of DCF. Fluorescence images were captured by a fluorescence microscope (Olympus IX51).

### 2.13 Chromatin immunoprecipitation

Chromatin immunoprecipitation (ChIP) was performed according to the ChIP Assay Kit (P2078) purchased from Beyotime Biotechnology. Briefly, immunoprecipitation assays were carried out by using aliquots of lysates containing 200 mg protein, and immunoprecipitation reaction was performed with anti‐H3K9me2 and anti‐IgG. ChIP‐enriched DNA analysed by qPCR with the primers in Table 1. Primers of TLR4 promoter designed for ChIP analysis were ranged from −2000 bp to +600 bp, which was obtained from www.ncbi.nlm.nih.gov/gene.

**TABLE 1:** ChIP‐qPCR primers

Promoter	Position	Sequence
P1	−1998 bp/−1473 bp	5′‐TGACTACCATTGCGTATCTT‐3′
		5′‐TCACATCTTCACCAACACTT‐3′
P2	−1529 bp/−904 bp	5′‐CAGATGCTGTGGAGAATCA‐3′
		5′‐GCTCTTAGAAGTGGAATCATAC‐3′
P3	−1026 bp/−548 bp	5′‐GAGGTATGTAAGGTAGAATGAG‐3′
		5′‐TCAAGGTGTCAGCAAGTG‐3′
P4	−617 bp/−97 bp	5′‐TCTAACTTCCTCTCCTGTGA‐3′
		5′‐ACTGGTGTCTTCTCTTCCT‐3′
P5	−117 bp/+591 bp	5′‐AAGACTCAAGAAGCCACAG‐3′
		5′‐TCACAGAGCCACAAGGTA‐3′

### 2.14 Dual‐luciferase reporter assay

HK2 cells were seeded in 24‐well culture plates at 8 × 10^4^ cells per well. Then, the cells were transfected with the luciferase report plasmid containing various fragments of TLR4 promoter (200 ng) together with the plasmids overexpressing LSD1 or the control plasmid (200 ng) in the presence of pRLTK (Renilla luciferase, 50 ng). After 24 h of transfection, the cells were lysed (E1941; Promega) and the luciferase activity was measured using the Dual‐Glo Luciferase Assay system (GloMax 20/20 Luminometer; E5311; Promega).

### 2.15 Statistical analysis

All results were indicated as mean ± standard error of the mean. Statistical analyses involved one‐way analysis of variance (anova) and the Student–Newman–Keuls test. *p* < 0.05 was considered as significant difference.

## 3 RESULTS

### 3.1 LSD1 was up‐regulated after renal IRI

**FIGURE 1:**
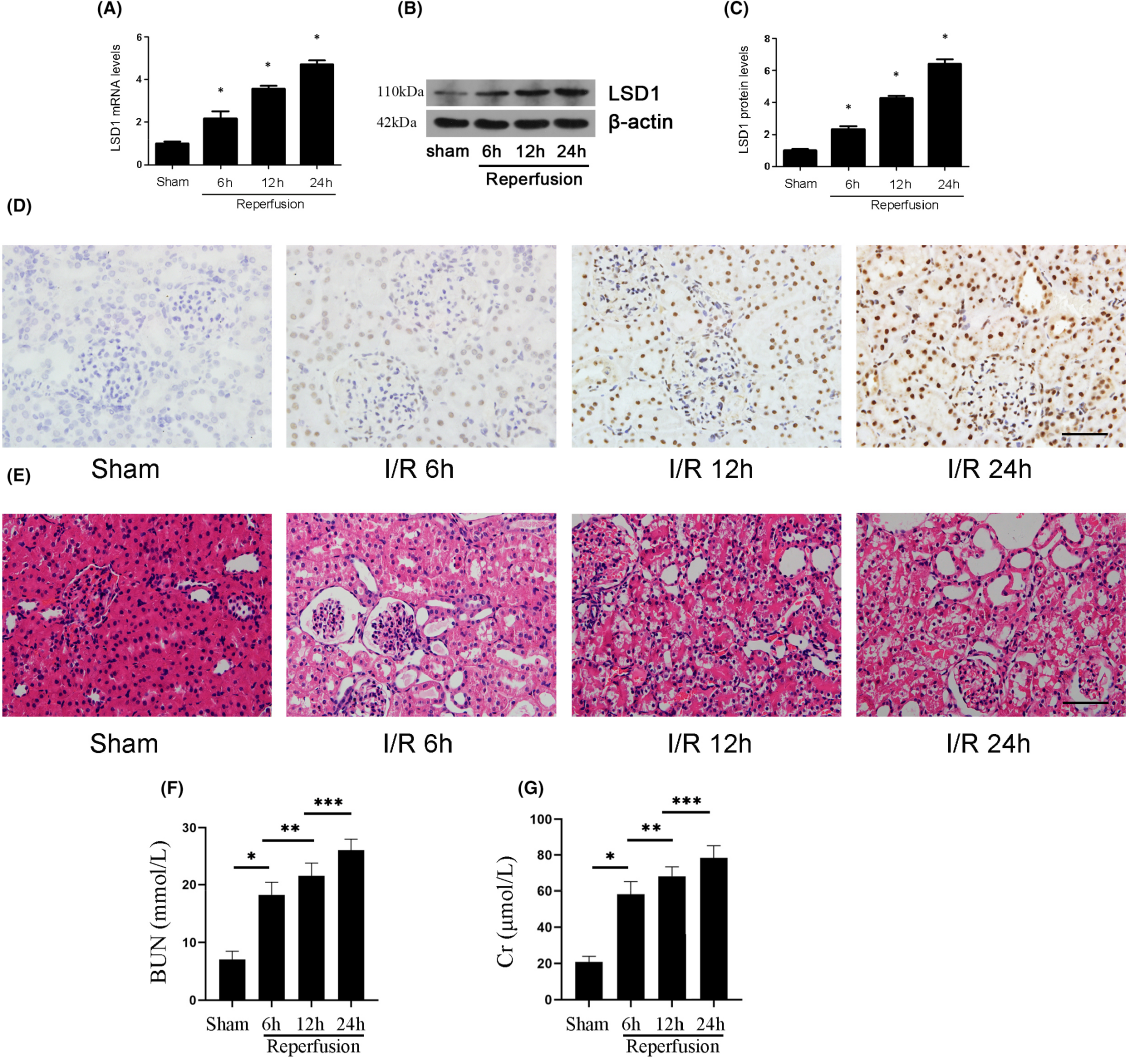
LSD1 expression was elevated in mice after renal IRI. All mice were subjected to ischaemia 30 min and reperfusion 6, 12 and 24 h, respectively. (A) LSD1 mRNA level was elevated after renal IRI. (B‐C) LSD1 protein level was elevated after renal IRI, and the quantification also was shown. (D) The immunohistochemical staining of LSD1 was examined in renal tissues at 6, 12 or 24 h of reperfusion time (×400; scale bars = 40 μm). (E) The morphological changes of the renal tissues detected by H&E staining at 6, 12 or 24 h of reperfusion (×400; scale bars = 40 μm)(F–G)The levels of Cr and BUN were detected after renal IRI (*n* = 8) The results were expressed as mean ± standard error of mean (SEM). *p < 0.05, when compared with the sham group. **p < 0.05, when compared with the IR 6 h group. ***p < 0.05, when compared with the IR 12 h group

To evaluate whether LSD1 regulated the progression of renal IRI, the expression of LSD1 was examined at 6, 12 and 24 h using WB and PCR (Figure 1A–C). With reperfusion time prolonged, its expression gradually increased and reached the peak at 24 h. Next, immunohistochemical staining showed that LSD1 mainly expressed in the nucleus and the highest expression was observed at 24 h (Figure 1D). Then, haematoxylin and eosin (H&E) staining was used to observe the morphological changes; the renal tissues of mice in sham group were basically normal. Nevertheless, extensive proximal tubular injury was shown in IRI group including loss of the brush border, dilation of renal tubules and oedema of the interstitium. Renal injury was aggravated with the prolongation of reperfusion time and reached peak at 24 h (Figure 1E). Furthermore, the levels of Cr and BUN were also increased after renal IRI, especially at 24 h (Figure 1F‐G). These results suggested that LSD might play an important role in the progression of renal IRI. Therefore, 24 h was chosen as reperfusion time in the following experiments.

### 3.2 LSD1 inhibitor alleviated renal IRI

**FIGURE 2:**
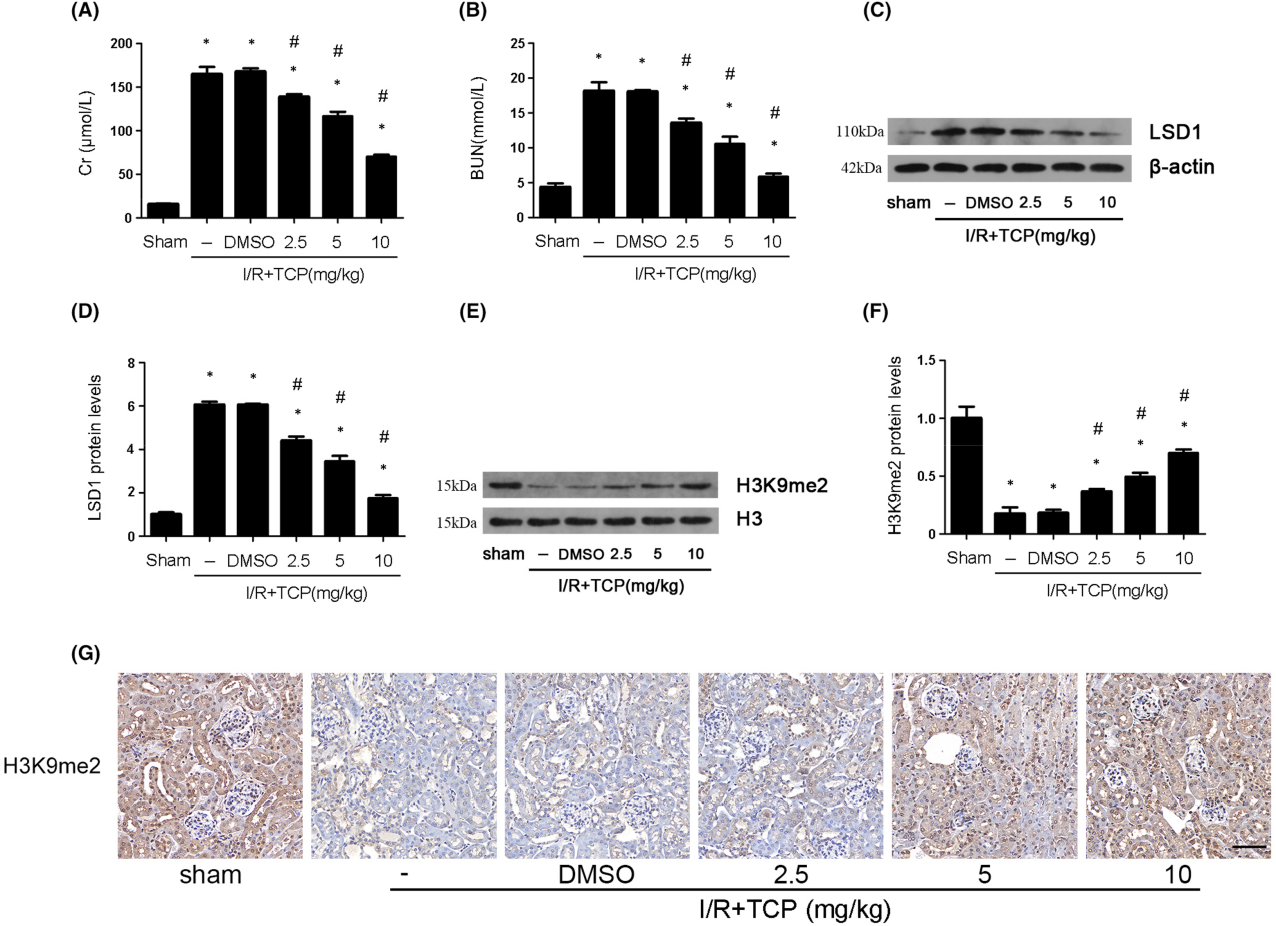
LSD1 inhibitor protected kidney tissue against IRI in mice. All mice were subjected to ischaemia 30 min and then reperfusion 24 h. (A–B) The effect of LSD1 inhibitor at various concentration (2.5, 5 or 10 mg/kg) on the levels of Cr and BUN in IRI group. (C–F) The effect of LSD1 inhibitor at various concentration on LSD1 and H3K9me2 expression in mice and the quantification. (G) Representative pictures of immunohistochemistry for H3K9me2 (×400; scale bars = 40 μm) (*n* = 8). The results were expressed as mean ± SEM. *p < 0.05, when compared with the sham group. #*p* < 0.05, when compared with the IRI group

To further explore the role of LSD1 in renal IRI, TCP, a well‐known specific inhibitor against LSD1, was employed. The levels of Cr and BUN were significantly reduced after the treatment of TCP (Figure 2A–B). Western blot showed that the treatment of TCP with the different concentration could inhibit the elevated expression of LSD1 induced by renal IRI (Figure 2C–D). Mice treated with TCP showed more obvious effects on renal function and the expression of LSD1 at the concentration of 10 mg/kg. LSD1 works mainly by removing monomethylation and dimethylation of H3K9me1/2. TCP inhibits LSD1 actively to protect the renal injury. Thus, we determined the effects of TCP on the expression of H3K9me2 in vivo by WB with different concentrations of TCP (0, 2.5, 5 and 10 mg/kg). The level of H3K9me2 was obviously down‐regulated in IRI group, which was reversed by TCP in a dose‐dependent manner, especially at the dose of 10 mg/kg (Figure 2E–F). The immunohistochemical results of H3K9me2 were consistent with the Western blot results (Figure 2G). In conclusion, these results suggested that TCP could significantly alleviate renal IRI by decreasing LSD1 expression and increasing H3K9me2 expression in a dose‐dependent manner. Therefore, the dose of 10 mg/kg was chosen in following experiment.

### 3.3 LSD1 inhibitor alleviated ferroptosis and oxidative stress induced by renal IRI

**FIGURE 3:**
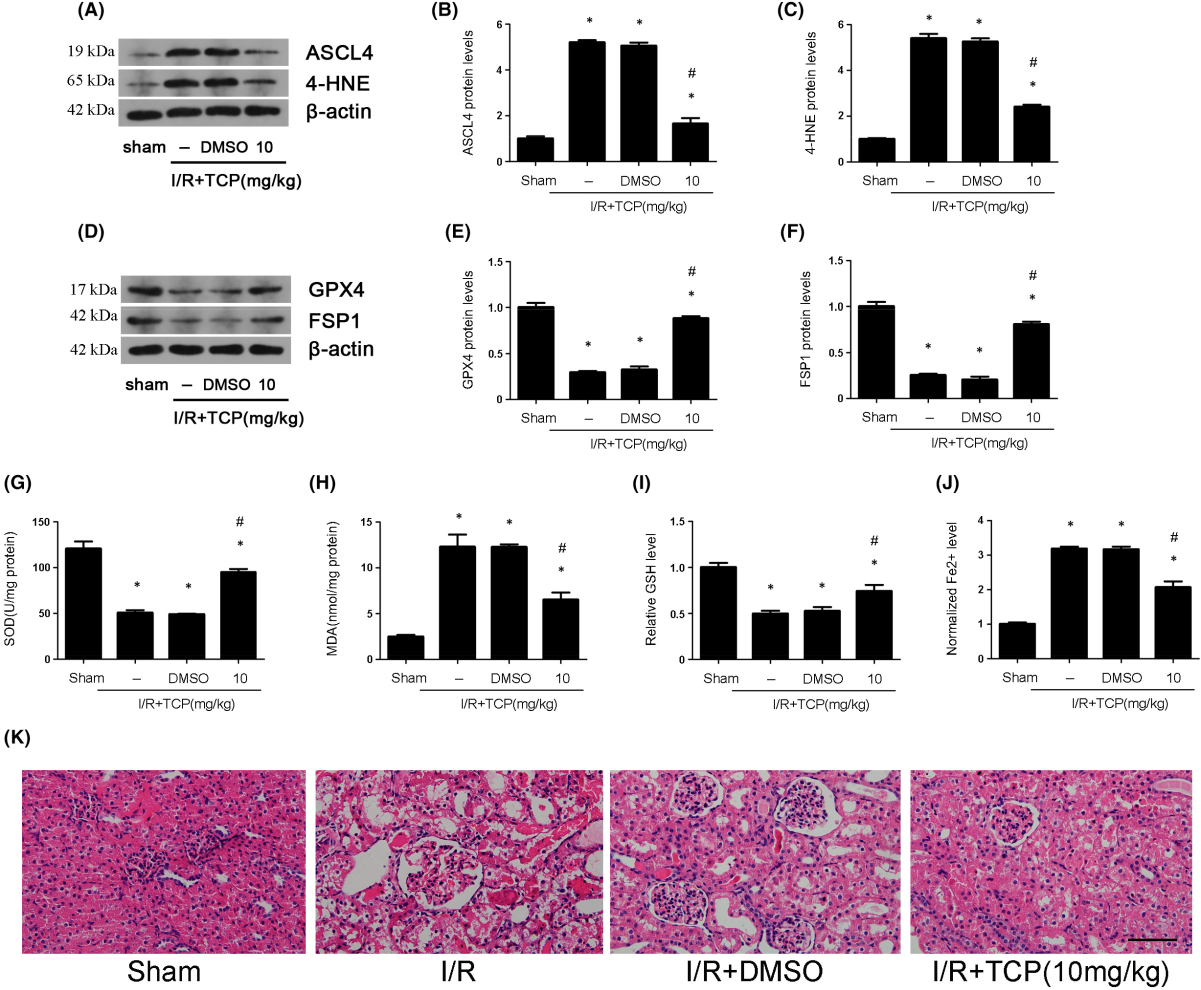
LSD1 inhibitor alleviated ferroptosis and oxidative stress that were caused by renal IRI in mice. All mice were subjected to ischaemia 30 min and then reperfusion 24 h. (A–F) The regulation of LSD1 inhibitor on the expression of ASCL4, 4‐HNE, GPX4 and FSP1 after renal IRI, and quantification was also shown. (G) The regulation of LSD1 inhibitor on the SOD activity in mice subjected to renal IRI. (H) The regulation of LSD1 inhibitor on the MDA content in mice subjected to renal IRI. (I) The regulation of LSD1 inhibitor on the GSH level in mice subjected to renal IRI. (J) The regulation of LSD1 inhibitor on the Fe^2+^ level in mice subjected to renal IRI. (K) The effect of TCP at the concentration (10 mg/kg) on renal structure damage detected by H&E staining (×400; scale bars = 40 μm) (*n* = 8). The results were expressed as mean ± SEM. *p < 0.05, when compared with the sham group. #*p* < 0.05, when compared with the IRI group

To investigate the effect of TCP on ferroptosis and oxidative stress in vivo, the following experiments were performed. The results showed that ASCL4 and 4‐HNE expression (Figure 3A–C), as well as MDA (Figure 3H) and Fe2+ level (Figure 3J), were elevated after renal IRI, which could be reversed by TCP treatment. However, the expression of GPX4 and FSP1 (Figure 3D–F), as well as SOD activity (Figure 3G) and GSH level (Figure 3I), was decreased after renal IRI and TCP could upregulate their expression. Besides, after the treatment of TCP, the kidneys of mice exhibited less tubular damage in the proximal tubules. TCP protected the kidneys by reducing dilatation of renal tubules, oedema of interstitium and infiltration of inflammatory cells in lumen (Figure 3K).

### 3.4 LSD1 level was increased during H/R In HK2 cells

**FIGURE 4:**
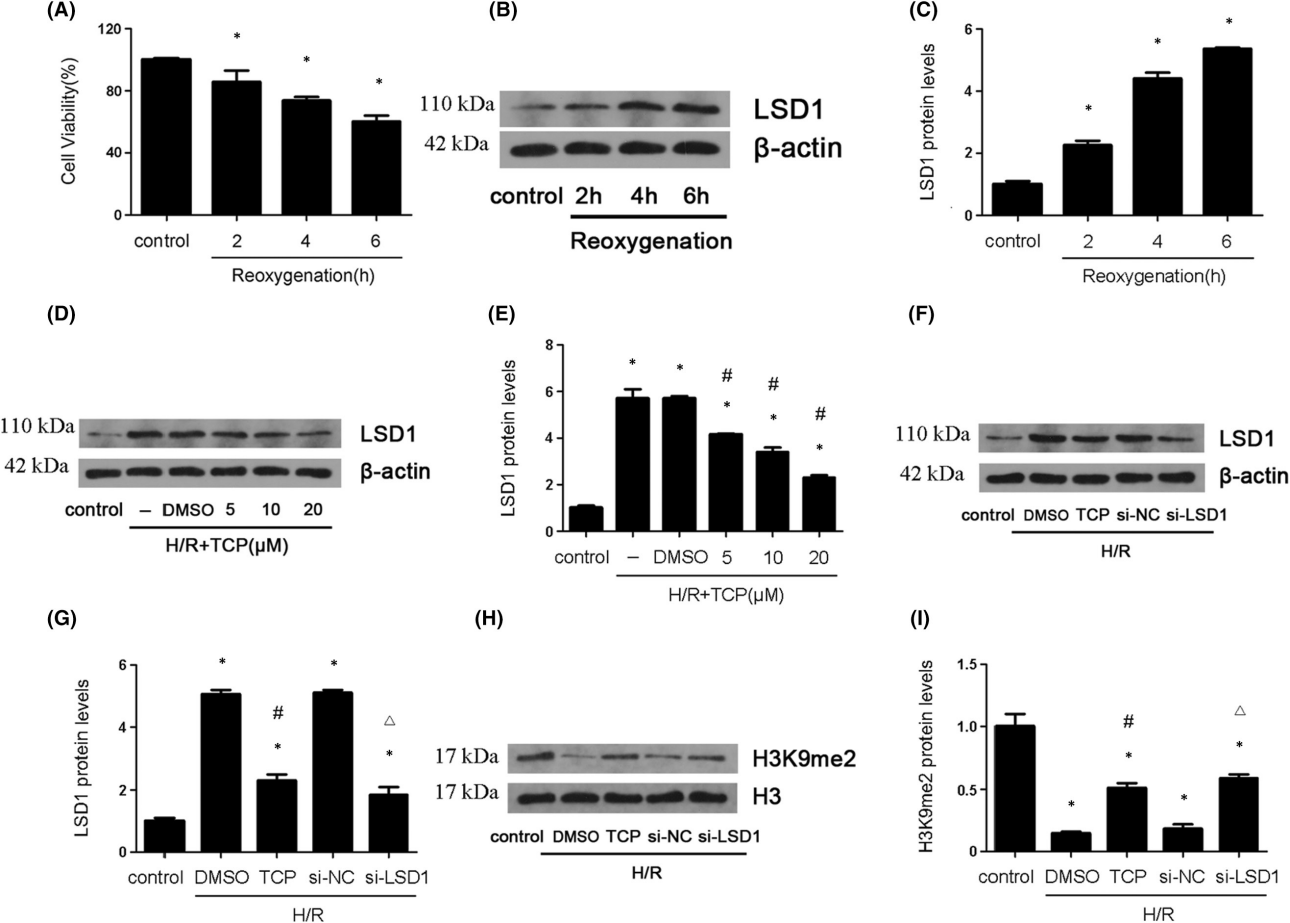
LSD1 expression were elevated during H/R induced injury in vitro. The HK‐2 cells were subjected to hypoxia 12 h and reoxygenation 2, 4 and 6 h. (A) The cell viability was examined by CCK‐8 kit in HK‐2 cells after H/R. (B–C) LSD1 expression was detected by Western blot in HK‐2 cells after H/R, and the quantification was also shown. (D–E) Various concentration of LSD1 inhibitor was employed to examine LSD1 expression, and the quantification was also shown. (F–I) The expression of LSD1 and H3K9me2 was examined by Western blot after inhibition of LSD1 with TCP or si‐RNA against LSD1 (*n* = 8). The results were expressed as mean ± SEM. *p < 0.05, when compared with the control group. #*p* < 0.05, when compared with the H/R group

Firstly, we investigated whether reoxygenation time affected cell viability and the expression of LSD1. The cell viability decreased gradually with the extension of the reoxygenation time and reached lowest point at 6 h (Figure 4A). Furthermore, LSD1 was elevated after H/R especially at reoxygenation 6 h (Figure 4B–C). Therefore, 6 h was chosen for following experiments. Next, we used different doses of TCP (5, 10 and 20 μmol/L) in vitro. TCP reduced the expression of LSD1 in a dose‐dependent manner (Figure 4D–E). Therefore, we chose 20 μmol/L as the optimal dose for subsequent experiments. In vitro, si‐RNA against LSD1 was used to genetically knock down LSD1. The expression of LSD1 was obviously down‐regulated by either TCP or genetic knockdown (Figure 4F–G). However, the level of H3K9me2 was opposite compared with the change of LSD1 expression (Figure 4H–I).

### 3.5 Suppression of LSD1 Decreased the expression of TLR4/NOX4 pathway, ferroptosis and oxidative stress in vitro

**FIGURE 5:**
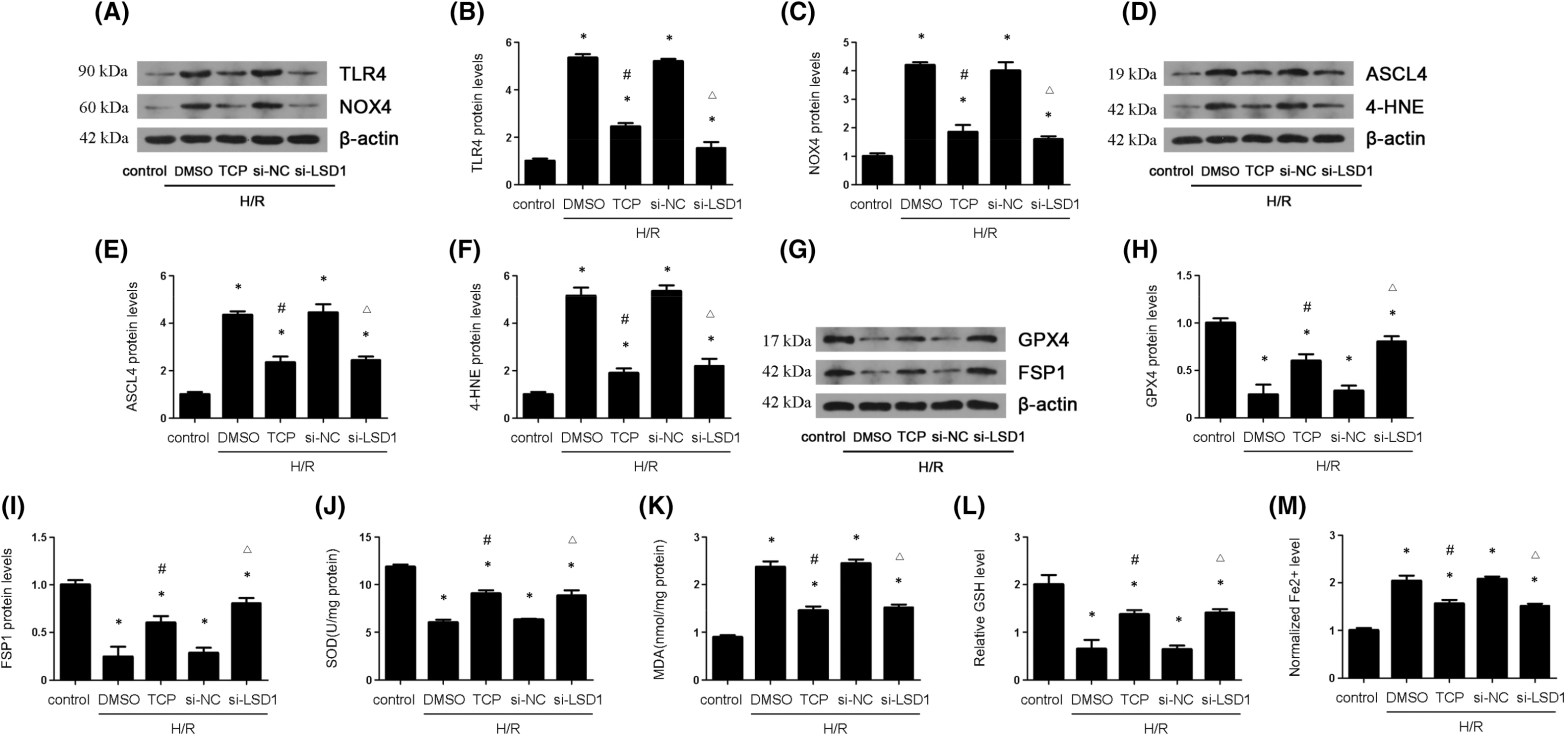
LSD1 inhibition decreased TLR4/NOX4, ferroptosis and oxidative stress that were caused by H/R in HK‐2 cells. The HK‐2 cells were subjected to hypoxia 12 h and reoxygenation 6 h. The cells were pretreated with TCP or transfected with si‐NC or si‐LSD1 for 24 h and then subjected to H/R. (A–C) The expression of TLR/NOX4 pathway was examined by Western blot, and the quantification was also shown. (D–I) The regulatory effect of LSD1 on ASCL4, 4‐HNE, GPX4 and FSP1 expression in HK‐2 cells after H/R, and quantification. (J) The regulation of LSD1 on SOD activity in HK‐2 cells after H/R. (K) The regulation of LSD1 on MDA content in HK‐2 cells after H/R. (L) The regulation of LSD1 on GSH level in HK‐2 cells after H/R. (M) The regulation of LSD1 on Fe^2+^ level in HK‐2 cells after H/R (*n* = 8). The results were expressed as mean ± SEM. *p < 0.05, when compared with the control group. #*p* < 0.05, when compared with the H/R + DMSO group. △*p* < 0.05, when compared with the H/R + si‐NC group

It was reported that TLR4/NOX4 signal pathway played a significant role in ferroptosis and oxidative stress. The results showed that TCP or LSD1 knockdown remarkably decreased the elevated TLR4 and NOX4 expression induced by H/R (Figure 5A–C). Subsequently, we investigated the proteins involved in ferroptosis and oxidative stress. ASCL4,4‐HNE (Figure 5D–F), MDA (Figure 5K) and Fe2+ (Figure 5M) were increased while GPX4, FSP1(Figure 5G–I), SOD (Figure 5J) and GSH (Figure 5L) were decreased after H/R process. However, all these changes were reversed by TCP or LSD1 knockdown. The results also revealed that treatment with TCP or LSD1 knockdown alleviated the elevation of ROS induced by H/R (Figure 5N). In brief, these results suggested that LSD1 activated TLR4/NOX4 pathway and promoted ferroptosis and oxidative stress in vitro.

### 3.6 The inhibition of TLR4/NOX4 reduced oxidative stress and ferroptosis

**FIGURE 6:**
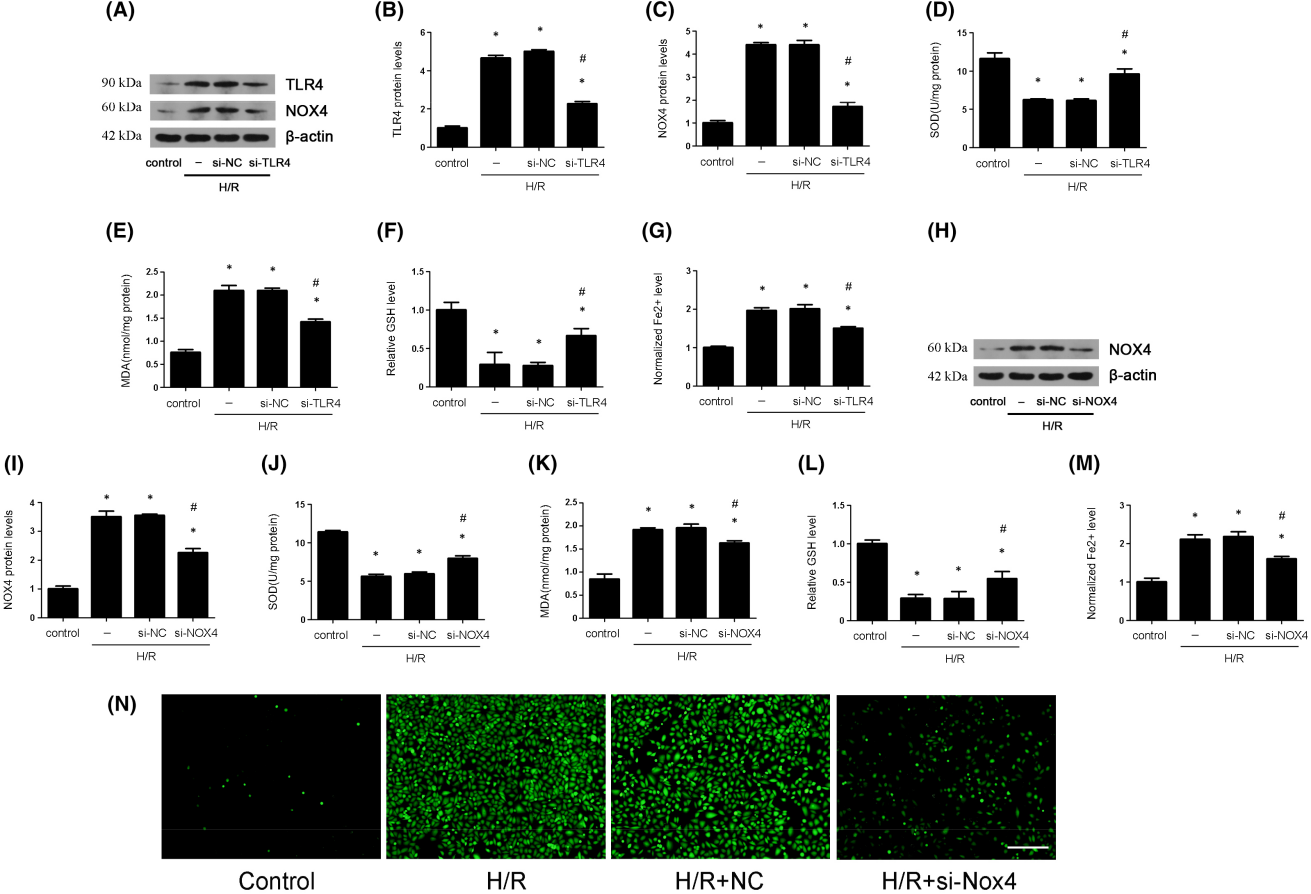
TLR4/NOX4 silence decreased ferroptosis and oxidative stress that were caused by H/R in HK‐2 cells. The HK‐2 cells were subjected to hypoxia 12 h and reoxygenation 6 h. The HK‐2 cells were transfected with si‐NC, si‐TLR4 or si‐NOX4 for 24 h and then subjected to H/R. (A–C) The expression of TLR/NOX4 pathway was examined by Western blot after TLR4 silence and the quantification was also shown. (D–G) The SOD, MDA, GSH and Fe^2+^ levels were examined after TLR4 silence in vitro. (H–I) The expression of NOX4 was examined by Western blot after NOX4 silence, and the quantification was also shown. (J–M) The SOD, MDA, GSH and Fe^2+^ levels were examined after NOX4 silence in vitro. (N) Reactive oxygen species (ROS) production was measured by 2′,7′‐dichlorodihydrofluorescein diacetate molecular probes (×100; scale bars = 100 μm) after NOX4 silence (*n* = 8). The results were expressed as mean ± SEM. *p < 0.05, when compared with the control group. #*p* < 0.05, when compared with the H/R group

To further investigate how TLR4/NOX4 affect ferroptosis and oxidative stress in renal IRI, we used si‐RNA against TLR4 and NOX4. The results revealed that si‐TLR4 could reduce TLR4 and NOX4 expression (Figure 6A–C), si‐NOX4 could reduce NOX4 expression (Figure 6H–I). Further, we found that either si‐TLR4 or si‐NOX4 could increase the reduced level of GSH and SOD, which were induced by H/R. However, we found that either si‐TLR4 or si‐NOX4 could reverse the elevated MDA and Fe2^+^ level (Figure 6D–G,J‐M). In addition, the level of ROS production in H/R cells was higher than in H/R cells transfected with si‐NOX4 (Figure 6N).

### 3.7 LSD1 regulated oxidative stress and ferroptosis through TLR4/NOX4 pathway

**FIGURE 7:**
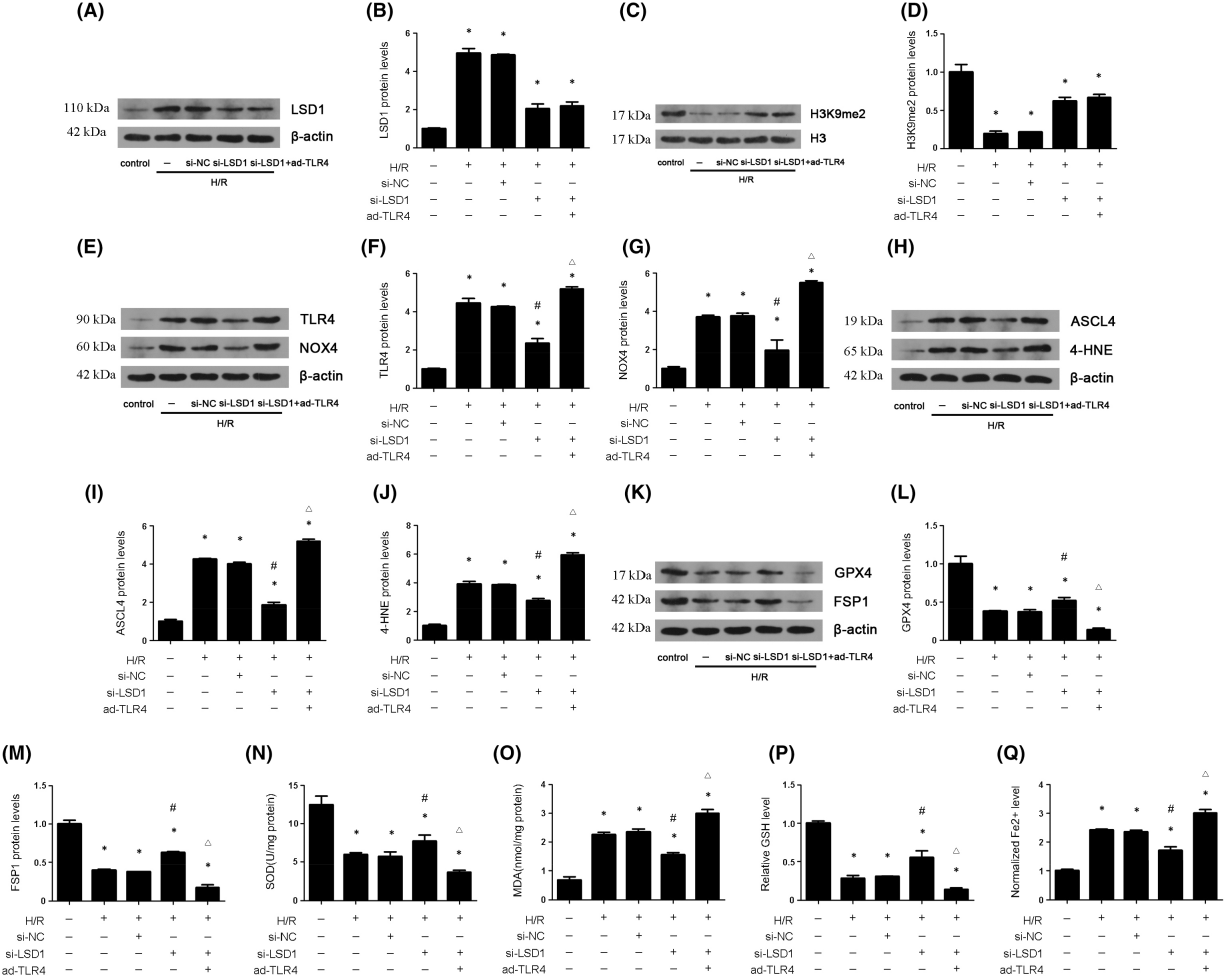
LSD1 aggravates ferroptosis and oxidative stress induced by H/R process via activation of TLR4/NOX4 pathway in HK‐2 cells. The HK‐2 cells were subjected to hypoxia 12 h and reoxygenation 6 h. The HK‐2 cells were transfected with si‐NC or LSD1 for 24 h, and then subjected to H/R, with or without infected with adenovirus carrying TLR4. (A–D) The expression of LSD1 and H3K9me2 was examined by Western blot after LSD1 silence with or without ad‐TLR4, and quantification was also shown. (E‐G) The expression of TLR4/NOX4 was examined by Western blot after LSD1 silence with or without ad‐TLR4, and quantification was also shown. (H‐M) The expression of ASCL4, 4‐HNE, GPX4 and FSP1 was examined by Western blot after LSD1 silence with or without ad‐TLR4, and quantification was also shown. (N‐Q) The SOD, MDA, GSH and Fe2+ levels were detected after LSD1 silence with or without ad‐TLR4, and quantification was also shown (n = 8). The results were expressed as mean ± SEM. *p < 0.05, when compared with the control group. #P < 0.05, when compared with the H/R + si‐NC group. △*p* < 0.05, when compared with the H/R + si‐LSD1 group

To further demonstrate how LSD1 regulated oxidative stress and ferroptosis during renal IRI, TLR4 reduction, mediated by inhibition of LSD, was compensated through delivering the adenovirus carrying TLR4 in vitro. The results showed that the compensation of TLR4 hardly affect the expression of LSD1 and H3K9me2 (Figure 7A‐D). Further, we found that the compensation of TLR4 blunted the reduction of TLR4, NOX4, ASCL4 and 4‐HNE expression induced by the LSD1 inhibition, as well as MDA and Fe2+ levels. Moreover, the compensation of TLR4 also reversed the elevation of GPX4 and FSP1 expression, as well as the level of SOD and GSH(Figure 7E‐Q).

### 3.8 LSD1 activated TLR4 transcriptional activity of TLR4 through removal of H3K9me2 from TLR4 promoter

**FIGURE 8:**
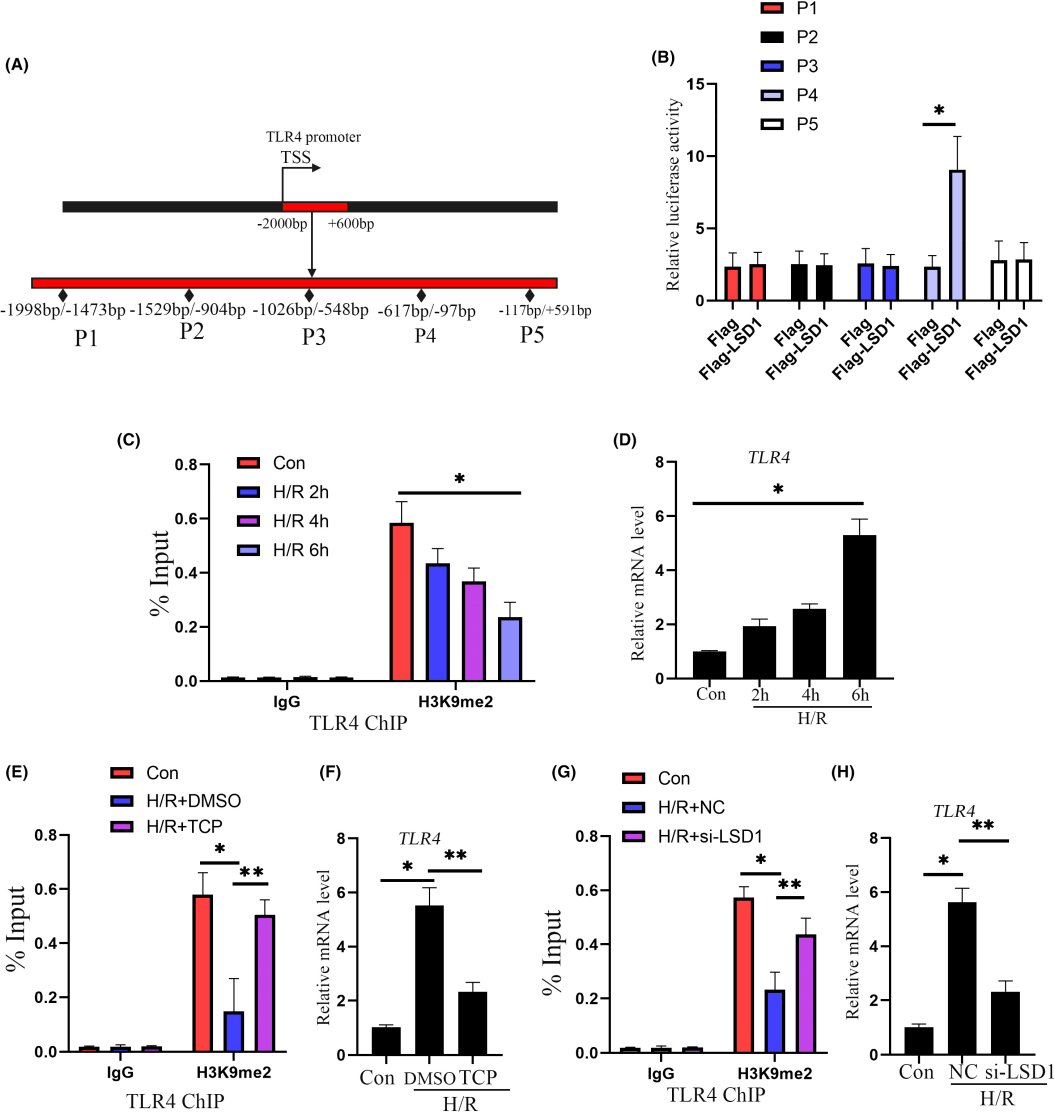
LSD1 activated TLR4 transcriptional activity of TLR4 through removal of H3K9me2 from TLR4 promoter. (A) Promoter sequence of TLR4 (from −2000 bp to +600 bp) was obtained from NCBI public database. (B) HK2 cells were transfected with indicated plasmids carrying Flag‐LSD1 and truncation of TLR4 promoter, and transcriptional activity of TLR4 promoter was detected by luciferase reporter assay. *p < 0.05, when compared with the Flag group. (C) The binding of H3K9me2 to TLR4 promoter was tested by ChIP assay. *p < 0.05, when compared with the con group. (D) mRNA level of TLR4 was assessed by qPCR. *p < 0.05, when compared with the con group. (E) ChIP assay was employed to evaluated the enrichment of H3K9me2 in TLR4 promoter. *p < 0.05, when compared with the con group. **p < 0.05, when compared with the H/R + DMSO group. (F) mRNA level of TLR4 was assessed by qPCR. *p < 0.05, when compared with the con group. **p < 0.05, when compared with the H/R + DMSO group. (G) The binding of H3K9me2 to TLR4 promoter was tested by ChIP assay. *p < 0.05, when compared with the con group. **p < 0.05, when compared with the H/R + NC group. (H) mRNA level of TLR4 was assessed by qPCR. *p < 0.05, when compared with the con group. **p < 0.05, when compared with the H/R + NC group

Promoter sequence of TLR4 was obtained from NCBI database (From −2000 bp to +600 bp). Flag‐tagged various truncation of TLR4 promoter plasmids were transfected into HK‐2 cells, and H/R model was established. As shown in Figure 8A–B, LSD1 enabled −619 bp/−97 bp promoter transcriptional activity of TLR4. Notably, H3K9me2 enriched in the TLR4 promoter upon normoxic condition (Figure 8C). Once stimulated by H/R challenge, H3K9me2 was erased from TLR4 promoter chromatin in a reoxygenation time dependent manner (Figure 8C). Accompanying these alterations, TLR4 transcriptional activity was elevated, as evidenced by up‐regulated mRNA level during H/R challenge. Simultaneously, inactivation of LSD1 with TCP remarkably enabled H3K9me2 to accumulate in TLR4 promoter, resulting in reduction of TLR4 mRNA level during H/R process (Figure 8E–F). In parallel, silencing LSD1 with si‐RNA efficiently promoted H3K9me2 to enrich in TLR4 promoter and contributed down‐regulation of TLR4 mRNA level (Figure 8G–H).

### 3.9 LSD1 inhibition decreased TLR4/NOX4 pathway in vivo

**FIGURE 9:**
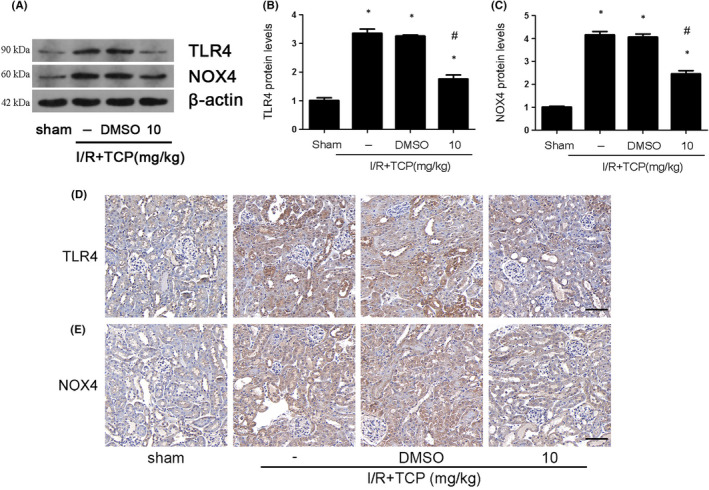
LSD1 inhibitor ameliorated TLR4/NOX4 activation that was induced by renal IRI in mice. All mice were subjected to ischaemia 30 min and then reperfusion 24 h. (A–C) The regulation of LSD1 inhibitor on TLR4/NOX4 expression in mice after IRI, and quantification was also shown. (D–E) Representative pictures of immunohistochemistry for TLR4 and NOX4 (×400; scale bars = 40 μm). (*n* = 8). The results were expressed as mean ± SEM. *p < 0.05, when compared with the sham group. #*p* < 0.05, when compared with the IRI group

Finally, we used TCP to confirm the effect of LSD1 on TLR4/NOX4 pathway in vivo. As the results showed, the level of TLR4 and NOX4 was elevated after IRI; however, TCP could decrease their expression (Figure 9A–C). The immunohistochemical results of TLR4 and NOX4 were consistent with the Western blot results (Figure 9D‐E). Taken together, these results demonstrated that LSD1 inhibition might decrease the TLR4/NOX4 pathway in vivo.

## 4 DISCUSSION

This study was aimed to investigating the effect of LSD1 during renal IRI and the underlying mechanism. Renal IRI model and H/R model were successfully established using mice and HK‐2 cells. The results revealed that LSD1 expression was significantly elevated after IRI. Inhibition of LSD1 attenuated tissue damage, renal dysfunction, oxidative stress and ferroptosis induced by IRI. Meanwhile, LSD1 reduced the expression of H3K9me2 in vivo and in vitro. Then, we used TCP and si‐LSD1 to further detect the regulatory effect of LSD1 on ferroptosis and oxidative stress through TLR4/NOX4 during H/R. Therefore, this study firstly suggested that LSD1 might be a target for treatment of renal IRI.

LSD1, a chromatin‐modifying enzyme, targets mono‐ or di‐methylated histone H3K4 and H3K9 to regulate gene expression as an activator or repressor.^20^ LSD1 plays a vital role in physiological function, including differentiation, development, thermogenesis and inflammation. Also, LSD1 participates in pathological processes, which was well‐known in tumorigenesis and development. Previous studies have found that LSD1 regulated organic IRI through different mechanism. It was reported that LSD1 mediated the crosstalk between histone methylation and phospholipid metabolism in myocardial IRI and suppression of LSD1 could afford cardioprotective effects against IRI.^10^ Other study showed that LSD1 expression was elevated in cerebral IRI, suggesting its important role in neuron function.^11^ However, regarding the existence and significance of LSD1 in the kidney, there is still limited. In this study, we found that both protein and mRNA levels of LSD1 increased significantly, both in vivo and in vitro. Furthermore, H3K9me2 was decreased in the process of IRI TCP and si‐LSD1could reduced LSD1 expression and protect renal tissue and HK‐2 cells against IRI or H/R. These results demonstrated that LSD1 had an important role in regulating renal IRI.

Ferroptosis, characterized by accumulated iron and serious lipid peroxidation, is a different form of regulated cell death. Ferroptosis has been found to participate in various disease.^21^, ^22^, ^23^ Meanwhile, in in vivo and in vitro model of renal IRI, ferroptosis was reported to aggravate tissue and cellular injury, whereas inhibition of ferroptosis could prevent against renal IRI.^24^, ^25^ Consistent with the previous studies, we found that ferroptosis was activated in renal IRI. The levels of ASCL4, 4‐HNE and Fe^2+^ were elevated in IRI and H/R, whereas the levels of GPX4, FSP1 and GSH were decreased. Further, the inhibition of LSD1 with TCP or si‐RNA could alleviate ferroptosis induced by IRI or H/R, which illustrated that LSD1 regulated ferroptosis caused by renal IRI.

Oxidative stress, which results from the accumulation of high concentration of ROS, plays an important role during the organic IRI.^26^ Besides, when iron is present in excess, it disrupts redox homeostasis and catalyses the spread of ROS, and thus leads to oxidative stress. Simultaneously, oxidative stress leads to ferroptosis as an iron‐dependent form of cell death.^27^ Previous studies indicated that oxidative stress took part in renal IRI through different mechanism.^28^, ^29^ Consistent with the previous, our study revealed that renal IRI induced oxidative stress as expected, as the SOD activity decreased and MDA content increased after IRI. Furthermore, we found that the inhibition of LSD1 with TCP or si‐RNA could reduce MDA and increase SOD level in vivo and in vitro, which indicated that LSD1 promoted renal IRI through modulating oxidative stress.

TLR4 is a member of Toll‐like receptor family that plays a crucial role in innate immunity and inflammation, as well as IRI.^30^ NADPH oxidase 4 (NOX4) is required in production of ROS and induces tissue damage during renal IRI.^31^

Previous study found that TLR4/NOX4 could promote cellular apoptosis and thus aggravated tissue injury.^32^ Consistent with previous studies, our study showed the expression of TLR4/NOX4 was elevated during renal IRI both in vivo and in vitro. Si‐RNA against either TLR4 or NOX4 could both reduce oxidative stress and ferroptosis level. Moreover, in order to demonstrate the relationship between LSD and TLR4/NOX4 pathway, LSD1 inhibitor or si‐RNA was employed. The results showed that both TCP and si‐LSD1 could alleviate the expression of TLR4/NOX4 and suppress oxidative stress and ferroptosis. To further demonstrate the role of TLR4/NOX4 in the regulation of LSD1 on oxidative stress and ferroptosis during renal IRI, we compensated the LSD1 inhibition induced TLR4 reduction through delivering the adenovirus carrying TLR4 to HK‐2 cells. The results showed that the compensation of TLR4 blunted the LSD1 inhibition mediated reduction of NOX4, ASCL4 and 4‐HNE expression, as well as MDA and Fe2+ levels. Moreover, the compensation of TLR4 also reversed the elevation of GPX4 and FSP1 expression, as well as the level of SOD and GSH. Besides, to illustrate the specific mechanism, we conducted ChIP assay to explore the action of LSD1 on TLR4. The results showed that LSD1 promoted −619 bp/−97 bp promoter transcriptional activity of TLR4 by removing H3K9me2 from the TLR4 promoter.

In conclusion, we demonstrated that LSD1 promoted renal IRI through elevation of ferroptosis and oxidative stress. Furthermore, we found inhibition of LSD1 could alleviate TLR4/NOX4 pathway to reduce oxidative stress and ferroptosis. Overall, our study firstly indicated LSD1 might be a powerful therapeutic target for renal IRI.

### 4.1 Clinical perspectives

While LSD1, a lysine‐specific demethylase 1, has been shown to regulate the pathogenesis of various kidney diseases, the role of LSD1 in renal ischaemia–reperfusion injury remains unknown.

The current study demonstrated that LSD1 aggravates renal oxidative stress and ferroptosis induced by ischaemia–reperfusion injury; this effect might be mediated by activation of TLR4/NOX4 signalling pathway.

Our study broadens insights into the molecular mechanisms implicated in renal ischaemia–reperfusion injury, suggesting that LSD1 is a potential therapeutic target.

## AUTHOR CONTRIBUTIONS

**Ruikang Feng:** Conceptualization (equal); data curation (equal); formal analysis (equal); validation (equal); visualization (equal); writing – original draft (equal). **Yufeng Xiong:** Conceptualization (equal); formal analysis (equal); investigation (equal); methodology (equal); validation (equal); writing – original draft (equal). **Qin Huang:** Conceptualization (equal); funding acquisition (equal); validation (equal); writing – original draft (equal). **Hao Liu:** Formal analysis (supporting); investigation (supporting); methodology (supporting); writing – review and editing (supporting). **Xiaojie Zhao:** Formal analysis (supporting); methodology (supporting); writing – review and editing (supporting). **Zhiyuan Chen:** Data curation (supporting); investigation (lead); writing – review and editing (supporting). **Hui Chen:** Investigation (supporting); project administration (supporting); writing – review and editing (supporting). **Xiuheng Liu:** Methodology (equal); resources (equal); software (equal); supervision (equal); validation (equal); writing – review and editing (equal). **Lei Wang:** Data curation (supporting); investigation (supporting); resources (equal); software (equal); supervision (equal); validation (equal); visualization (equal); writing – review and editing (equal). **Xiaodong Weng:** Data curation (equal); writing – original draft (equal). **Yourong Lei:** Data curation (equal); writing – original draft (equal).

## CONFLICT OF INTEREST

The authors declare that they have no conflict of interests.

## Data Availability

The authors confirm that the data supporting the findings of this study are available within the article and its supplementary materials.
